# Restoring Esthetics after Anterior Tooth Loss for a Five-Year-Old Child: Natural Tooth Pontic Fiber Reinforced Prosthesis

**DOI:** 10.1155/2013/215816

**Published:** 2013-09-25

**Authors:** Dhirja Goel, Gaurav Kumar Goel

**Affiliations:** ^1^Department of Pedodontics and Preventive Dentistry, School of Dental Sciences, Sharda University, Plot No. 32-34, Knowledge Park-III, Greater Noida 201306, India; ^2^Dentessence Dental Care, Noida, India

## Abstract

The loss of anterior teeth can be hurtful to the patient both psychologically and socially. As the children are becoming more aware these days, they have also become conscious about their looks. Early loss of deciduous anterior teeth has a psychological effect on many children. In such young patients, paper replacement of the teeth can minimize these concerns. Many approaches have been described for this temporary replacement. This article describes the technique to use extracted natural teeth as pontics bonded to adjacent teeth with fiber reinforced resin. A fiber reinforced temporary replacement of missing teeth provides adequate strength and esthetic requirements in such cases.

## 1. Introduction

Loss of anterior teeth has a far-reaching impact on an individual's psyche. When it comes to children, it becomes all the more important. These days children are more aware of their surroundings and sometimes become very conscious about the loss of their deciduous teeth. In such patients, fiber reinforced composite resin based prosthesis may be a good replacement option [1–4].

The reinforcement of composite resins by fibres improves both their fracture toughness and resistance [5]. Glass fiber reinforced composite material offers a restorative alternative that produces minimally invasive, esthetic, and cost-effective metal-free tooth replacement. Saving time, ease of application, absence of metal allergy, and ease of cleaning are other advantages of this technique.

This case report describes the use of extracted natural deciduous teeth as pontics for a fibre reinforced composite resin Fixed partial denture.

## 2. Case Report

**Figure 1:**
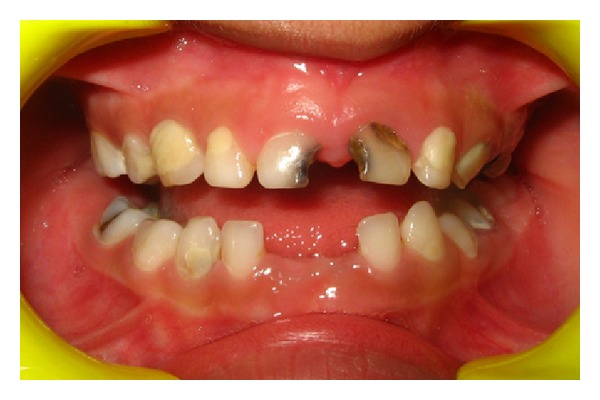
Preoperative condition.

A mother brought her five-year-old daughter with a complaint of pain in maxillary anterior deciduous teeth. Upon examination, it was seen that both the deciduous central incisors had deep cavities (Figure 1). An RVG showed deep caries in the teeth and almost complete resorption of roots. The teeth needed to be extracted but the child was very conscious of her looks and had to attend a family function few days later. She was not ready for extraction unless she was promised a replacement for missing teeth.

Following clinical and radiographic examinations, the decision was made to create a fixed partial denture reinforced with fiber reinforced composite using the natural teeth as a pontics.

**Figure 2:**
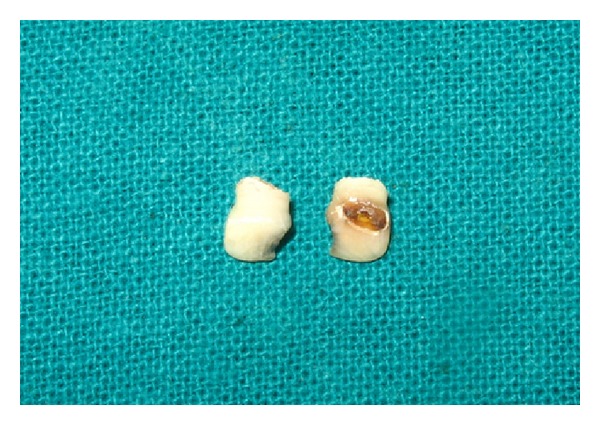
Extracted teeth.

**Figure 3:**
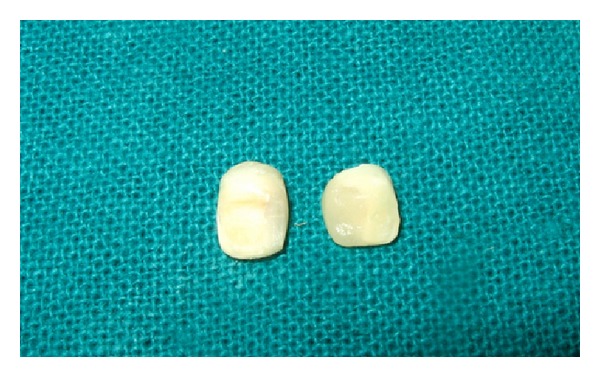
Natural teeth restored with composite to be used as pontics.

Both maxillary deciduous central incisors were extracted (Figure 2). The caries were removed with a high speed air turbine using diamond points and the teeth were restored to proper shape with composite resin (Z250, 3M ESPE, USA). The pulp chambers were also cleaned and filled with composite resin (Figure 3).

**Figure 4:**
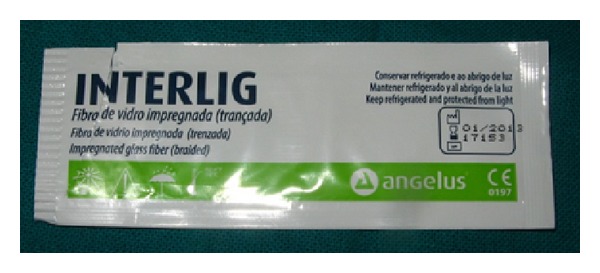
Interlig (fibre impregnated with composite resin).

**Figure 5:**
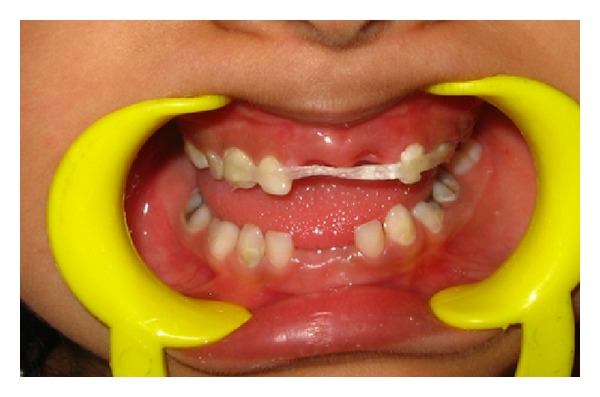
“Interlig” bonded to grooves in abutment teeth.

**Figure 6:**
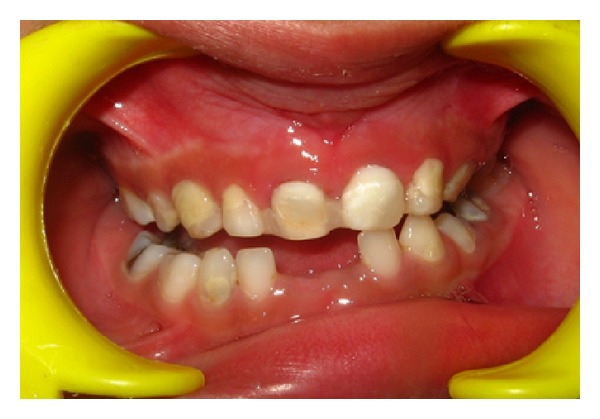
Natural teeth pontics bonded to “Interlig.”

**Figure 7:**
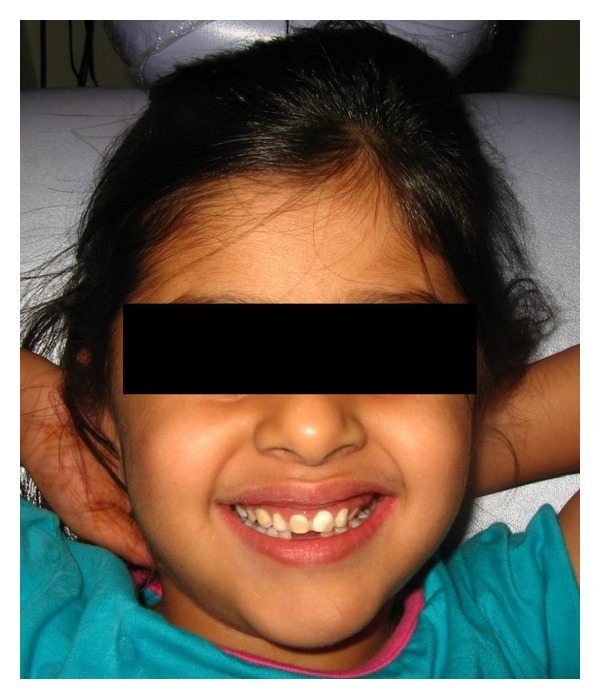
Final result.

A 2 mm wide horizontal groove was made on the labial surface of the deciduous left and right lateral incisors. Following completion of etching and bonding procedures, a thin layer of flowable composite resin was applied (without curing) to the labial groove and interproximal surfaces of the abutment teeth, width a length of 2 mm wide Composite reinforced fibre (Interlig, Angelus (Figure 4)) was placed on the surface of the teeth, and slight pressure was applied with a hand instrument to create close contact at the interproximal area. The excess resin composite was removed, and the fibre was light-cured for 20 s (Figure 5). The surface of the pontic was then prepared for bonding. A thin layer of flowable composite was applied to the natural tooth pontic, which was placed in the desired position on the interlig fibre and cured for 20 s (Figure 6). The patient's occlusion was checked for premature contacts, and the resin composite was polished using a polishing disc. The patient was happy with the final result (Figure 7).

## 3. Discussion

This clinical report describes the use of a fiber reinforced natural teeth prosthesis to restore aesthetics in a young child. The loss of anterior teeth can be psychologically and socially harmful to the patient, the trauma of which can be minimized by immediate replacement of teeth, preferably using fixed prostheses [6]. A conventional fixed prosthesis is not indicated in such cases. A removable partial denture is usually unacceptable to the patients as it is uncomfortable and needs patient compliance [7]. Also, it requires additional laboratory steps.

Thus the use of fiber reinforced prosthesis can be a good choice in such cases. It provides enhanced esthetic concept [7], eliminates need for complicated laboratory steps, and can be easily performed as a chairside replacement. The use of natural teeth as pontics helps in achieving better esthetics and strength as compared to acrylic or composite teeth. Also, natural teeth provide a more suitable surface for bonding of the fibre reinforced composite. 

The type of prosthesis described is an optimal method to restore esthetics in anterior tooth loss.
